# Noninvasive Measurement of EKG Properties of 3D Artificial Heart Muscle

**DOI:** 10.3934/celltissue.2017.1.12

**Published:** 2017

**Authors:** Betsy H. Salazar, Kristopher A. Hoffman, Anilkumar K. Reddy, Sridhar Madala, Ravi K. Birla

**Affiliations:** 1 Department of Biomedical Engineering, University of Houston, Houston, TX, USA; 2 Department of Medicine, Baylor College of Medicine, Houston, TX, USA; 3 Indus Instruments, Webster, TX, USA

**Keywords:** cardiovascular tissue engineering, cardiac construct, conduction velocity, electrical impulse mapping

## Abstract

Developing and testing a custom fabricated 16-electrode noninvasive direct contact system was necessary to assess the electrical properties of bioengineered heart muscle and to further evaluate the efficacy of cardiac constructs. By culturing neonatal rat primary cardiac cells on a fibrin gel, we constructed 3D artificial heart muscle (3D-AHM), as described in previous studies, which were used in validating this novel system. Electrical and mechanical functional assessment of the tissues was performed, which yielded contractile forces of the tissues, electrical field potential characteristics, and tissue conduction velocities (CV) (20–170 cm/s). Immunohistological evaluation revealed the formation of cardiac tissue structures and cardiomyocyte proliferation. EKG data analysis also yielded time delays between signals in the range of 0–38 ms with electrical maps showing some evidence of synchronous contraction within the fabricated tissues. This study demonstrates the effectiveness and practicality of our novel EKG measuring system to acquire distinct electrical metrics of 3D-AHM, which will aid in increasing the viability and applicability of cardiac tissue constructs.

## 1. Introduction

Treatment of heart failure remains a major medical challenge in the US and around the globe. Although several treatments modalities exist for patients with heart failure, heart transplant remains the most effective mitigator. However, there exists a concerning deficit of organs for transplantation; the number of adults on the waiting list for heart transplants has increased by 34.2% from 2003 to 2013 [1]. With organ donors in such high demand, it is crucial that alternative treatment paradigms are developed.

Tissue engineering focuses on the development of biological substitutes that serve in restoring, replacing, or improving the function of damaged tissues [2]. More specifically, cardiac tissue engineering examines the development of functional myocardium used to improve the lost functionality of the infarcted myocardium, in addition to modeling the physiology of cardiac development and diseases *in vitro* [3].

Based on current state of the art, functional cardiac constructs can be fabricated with a myriad of approaches including employing the use of biodegradable gels, polymeric scaffolds, and self-organization organization techniques [4–7]. In more detail, Radisic et al. used highly porous collagen scaffolds along with neonatal rat ventricular heart cells to develop engineered cardiac tissues [5]. Bursac et al. seeded neonatal rat ventricular cells on polyglycolic acid (PGA) scaffolds to fabricate cardiac muscle constructs [4]. Shimizu et al. utilized a self-organization strategy to construct three-dimensional cardiac constructs by stacking chick embryonic cardiomyocyte cell sheets on top of each other using a thermal sensitive polymer; yielding heart-like tissue constructs [6]. We have also developed heart muscle and other cardiovascular components [8–13]. Such progress includes, 3D-AHM that was formed using primary cardiac myocytes utilizing two different methods (layering and embedding) in which both methods showed functional tissue development [11]. In a separate study, cardioid tissue was produced using PDMS and laminin coated plates to culture neonatal cardiomyocytes [8].

As our group and others researchers continue to advance models for heart muscle and other tissues, it becomes vital to create systems with the ability to accurately assess their functionality. It is important to fabricate cardiac constructs that closely resemble native tissue in structure and performance. The intricate ansiotropic structure of the native cardiac muscle drives the electrical activity within the heart [14]. It is these electrical impulses that regulate the cardiac cycle and maintain sphygmic synchronisity. Understanding the electrical characteristics of cardiac constructs developed in our laboratory is crucial to properly replicate the functional attributes of native tissue. Here, we address the need of such characterization by developing a novel 16 electrode non-invasive system to record the electrical potentials of 3D-AHM. The results of this research can lead to improvement in our methodology and therefore in the viability of the constructs.

Several systems have been used in the past by other researchers and within our laboratory to assess the electrical properties of cardiac constructs [4,15,16]. One research group used a custom fabricated cylindrical plexiglass chamber fitted into an electrically grounded brass casing. This system used 8 microelectrodes placed 1.5mm to 5mm away from the site of stimulation [4]. Other groups have used the commercially available MEA1060 amplifier from Multi Channel Systems (Reutigen, Germany). This system is composed of 60 channels that record the EKG signals produced by cardiac constructs [15,17,18]. We developed a 32 channel direct contact system used with 3D-AHM constructs anaolgus to our present study [16]. This 32 channel direct contact system proved efficient in gathering all desired metrics, however, our goal is to move toward less invasive sensing systems as tearing of the constructs was seen at the tissue electrode interface; the result of the affixed gold plated pins penetrating the construct surface and therefore diminishing the viability of our tissues.

For this study, we developed a noninvasive direct contact system to assess the electrophysiological properties of 3D-AHM. First, we fabricated our cardiac constructs by seeding rat neonatal primary cardiac cells on a fibrin gel. Remodeling of the cells and integration at the cell-scaffold level lead to the formation of functional tissues. Second, we fabricated a custom 16 channel noninvasive electrode board compatible with the RHD2000-Series Amplifier Evaluation System (Intan Technologies, Los Angeles, CA). With this system we were able to produce electrical maps of the impulse propagation, calculate CVs, and acquire other metrics associated with the electrical properties of our heart muscle constructs.

## 2. Materials and Methods

The Institutional Animal Care and Use Committee (IACUC) at the University of Houston approved all animal protocols in accordance with the “Guide for the Care and Use of Laboratory Animals” (NIH publication 86–23, 1986).

### 2.1. Isolation of Primary Cardiac Cells

Neonatal primary cardiac cells were isolated from the hearts of 2 to 3 day old Sprague-Dawley rats employing methods previously established [9]. Each heart was divided into 3 to 4 pieces and placed in an ice-cold PBS phosphate buffer comprised of 116 mM NaCl, 20 mM HEPES, 5.5 mM glucose, 5.4 mM KCl, 1 mM Na_2_HPO_4_, and 0.8 mM MgSO_4_. After the blood cells were gently rinsed, the pieces were transferred to a second phosphate buffers solution for additional mincing. Tissues were cut into 1 mm^2^ fragments and placed in a dissociation solution (DS) consisting of 0.32 mg/mL collagenase type 2-filtered (Worthington Biochemical Corporation, Lakewood, NJ) and 0.6 mg/mL pancreatin in phosphate buffer. The tissue sections and 15mL of DS were placed in an orbital shaker for 30 minutes at 37°C and 60 rpm to carry out serial digestion. After the 1^st^ digestion was completed, the supernatant was collected in 3 mL of horse serum to neutralize the enzyme, and placed in a centrifuge for 5 min at 1000 rpm and 4°C. The cell pellet was re-suspended in 5 mL of horse serum and maintained in an incubator at 37°C, supplied with 5% CO_2_. Fresh DS was added to the partially digested tissue and the digestion process was repeated 2 to 3 times. The acquired cells were then pooled, centrifuged and suspended in culture medium (CM). The CM used consists of M199 (Life Technologies, Grand Island, NY) along with 20% F12K (Life Technologies), 10% fetal bovine serum, 5% horse serum, 1% antibiotic-antimycotic, 40 ng/mL hydrocortisone, and 100 ng/mL insulin. Trypan blue (4%) staining, was performed according to the manufacturer’s protocol to analyze cell viability.

### 2.2. Culture Plates Preparation

All 35 mm tissue culture plates were coated with 2 mL of SYLGARD (PDMS, type 184 silicone elastomer) (Dow Chemical Corporation, Midland, MI). The plates were air dried for 2 weeks allowing the silicone coating to form properly. Once the coating had cured completely, the plates were sterilized with 80% ethanol before use. 4 minutien pins (Fine Science Tools, Foster City, CA) 0.1 mm in diameter, were placed into the PDMS coating of the culture plate to serve as anchor points and form a 20 mm × 20 mm square.

### 2.3. Fabrication of Cardiac Muscle

The 3D-AHM was fabricated using a fibrin gel and isolated primary cardiac cells. Fibrin gel was formed by adding 1mL of CM containing 10 U/mL thrombin to the PDMS coated surface of each culture plate. Subsequently, 500 μL of saline containing 20 mg/mL fibrinogen was added. Fibrinogen is cleaved and cross-linked by exposure to thrombin, yielding an insoluble fibrin scaffold ideal for construct fabrication [7]. Once both solutions had been added, the culture plates were shaken well to ensure complete mixing and complete plate coverage and placed in the incubator for 45 minutes to stimulate the formation of the gel. Primary cardiac cells, isolated from the entire neonatal rat hearts, were diluted in CM at 2 million cells/mL and 2 mL of the solution were added to each plate with fibrin gel. The cells were cultured in an incubator at 37°C supplied with 5% CO_2_; media was changed every 2 days. This process resulted in 3D tissue with dimensions of 20 mm × 20 mm and a thickness of ~ 200–300 μm.

### 2.4. Contractile Properties Assessment

After 3D-AHM was formed, contracting synchronously (5–6 days in culture), contractile forces were measured using a highly sensitive TRI202PAD micro-force transducer (Panlab, Barcelona, Spain). In efforts to avoid damage to the construct and minimize the amount of force lost due to vibration, a thin rigid wire was used to attach the tissues to the force transducer. The output signals from the force transducer were processed using the PowerLab16/35 data acquisition system (ADInstruments, Colorado Springs, CO), and then transmitted to LabChart for analysis where the peak analysis feature was used to calculate the maximum twitch force of the tissue constructs.

### 2.5. Immunohistological Assessment

Heart muscle constructs were directly fixed in ice-cold acetone for 10 minutes. Nonspecific epitope antigens were blocked by using 10% goat serum for 1 hour at room temperature. The tissue segments were then incubated with mouse anti-α-actinin monoclonal antibody (1:200, Sigma, A7811), rabbit anti-collagen type I (1:100, Abcam, ab34710), and rabbit anti-connexin 43 (1:100, Abcam, ab11370) for 2 hours at room temperature. Next, the tissue fragments were treated with both goat anti-mouse and goat anti-rabbit secondary antibodies 1:400 (Alexa Fluor 488 and Alexa Fluor 546; Life Technologies, Grand Island, NY) for 1 hour at room temperature. Nuclei were counterstained with 2.5 μg/mL 4,6-diamidino-2-phenylindole (DAPI) at room temperature for 5 minutes. Lastly, the tissue samples were placed on VWR^®^ Microslides and fluorescent images were produced using a Nikon C2+ confocal laser-scanning microscope (Nikon Instruments Inc., Melville, NY).

### 2.6. Design and Fabrication of Noninvasive EKG Measuring System

**Figure 1.**
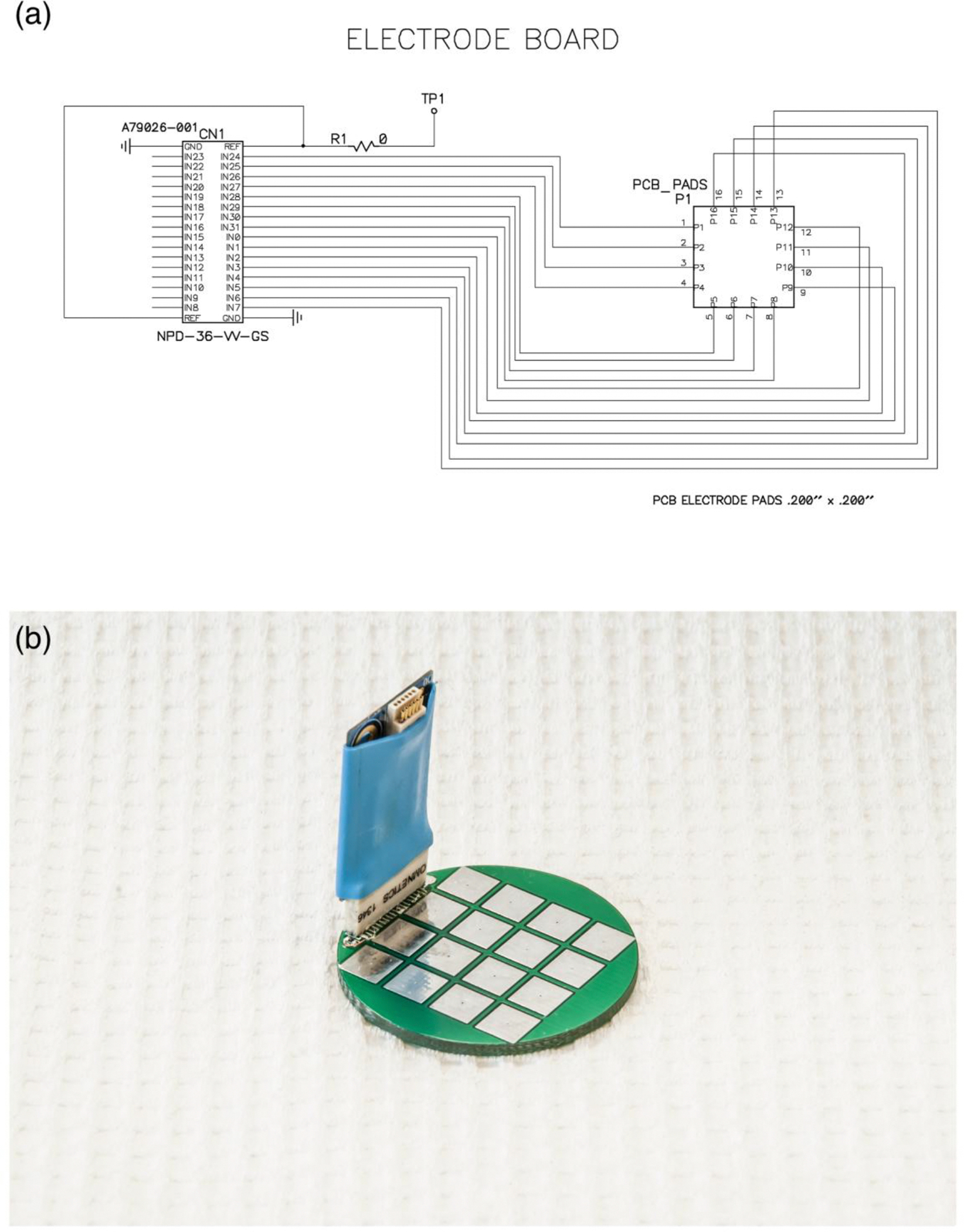
Printed circuit board design and electrode board. **(a)** Circuit schematic for the custom 16-electrode board design; **(b)** Image of printed circuit board with amplifier board from Intan Technologies attached using an Omnetics connector.

The RHD2000-Series amplifier evaluation system (Intan Technologies, Los Angeles, CA) was purchased. This highly customizable system includes an Opal Kelly XEM 6010 USB/TPGA interface module, which is capable of supporting up to 256 low noise amplifier channels with sampling rates that range from 1 to 30 kS/s. For this study we used a sampling rate of 5 kS/s and a bandwidth between 0.09 Hz and 1.00 kHz. Additionally, we designed and fabricated a custom 33 mm diameter circular printed circuit board (PCB) with 16 electrodes (5 mm × 5 mm square size each) (Figure 1). The PBC material used was standard FR-4 (glass reinforced epoxy laminate) at 1.6 mm thick. All 16 electrodes were placed on the top-side of the PBC with thin copper conductive traces connecting them on the bottom side to a standard Omnetics connector that is compatible with our bio-potential amplifiers (Figure 1). The 33 mm diameter PBC fit within a standard 35 mm tissue culture plate. For our initial testing the electrodes were tinned (Figure 1). In the future, an immersion gold process (ENIG) can be used for better biocompatibility if needed.

### 2.7. Testing of EKG Measuring System

**Figure 2.**
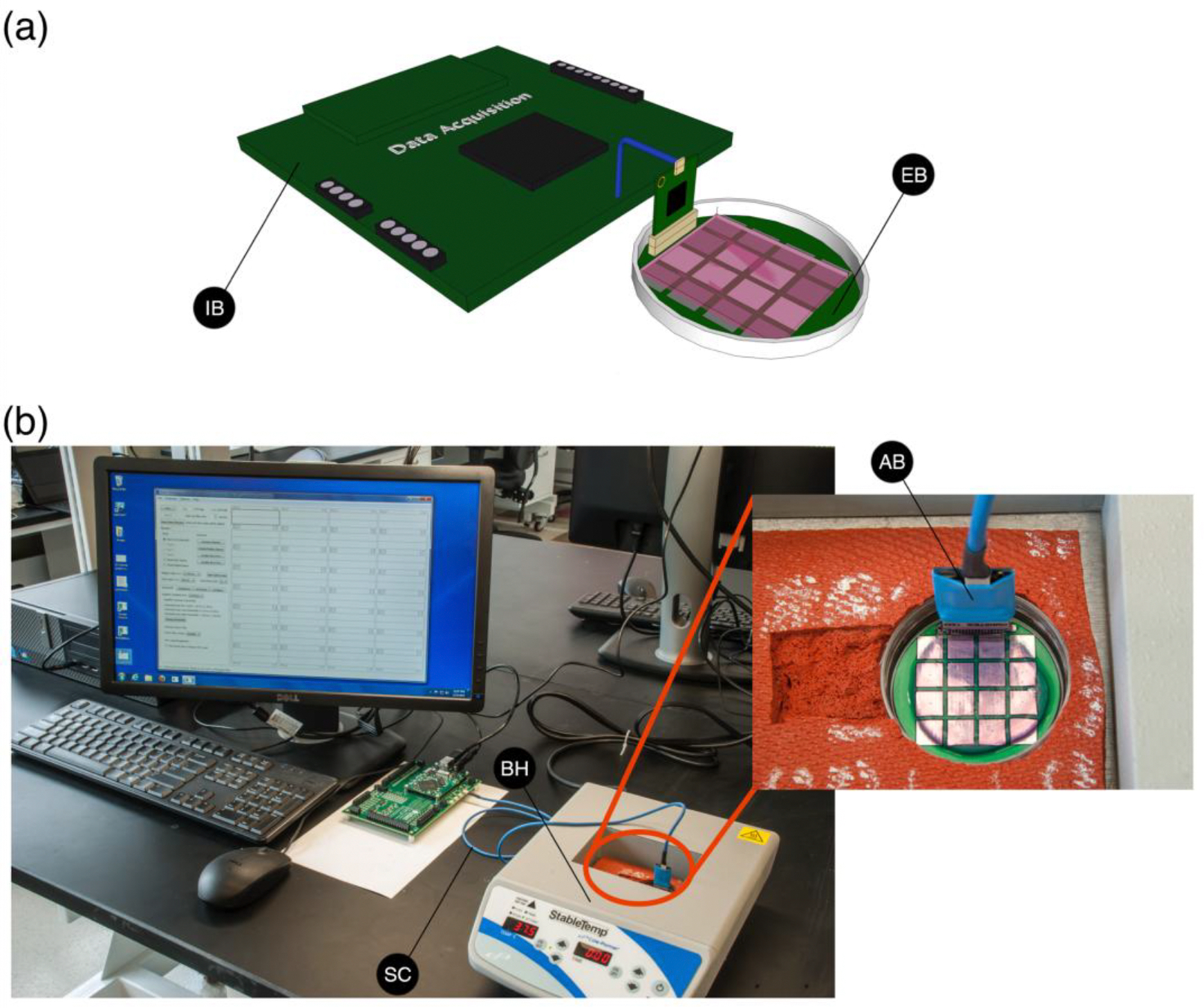
EKG acquisition system setup. **(a)** Schematic showing the data acquisition system, custom board and formed tissue construct. IB: interface board and EB: evaluation board; **(b)** Image showing the setup used to measure the electrical properties of the construct. SC: serial peripheral cable, BH: block heater, and AB: amplifier board.

**Figure 3.**
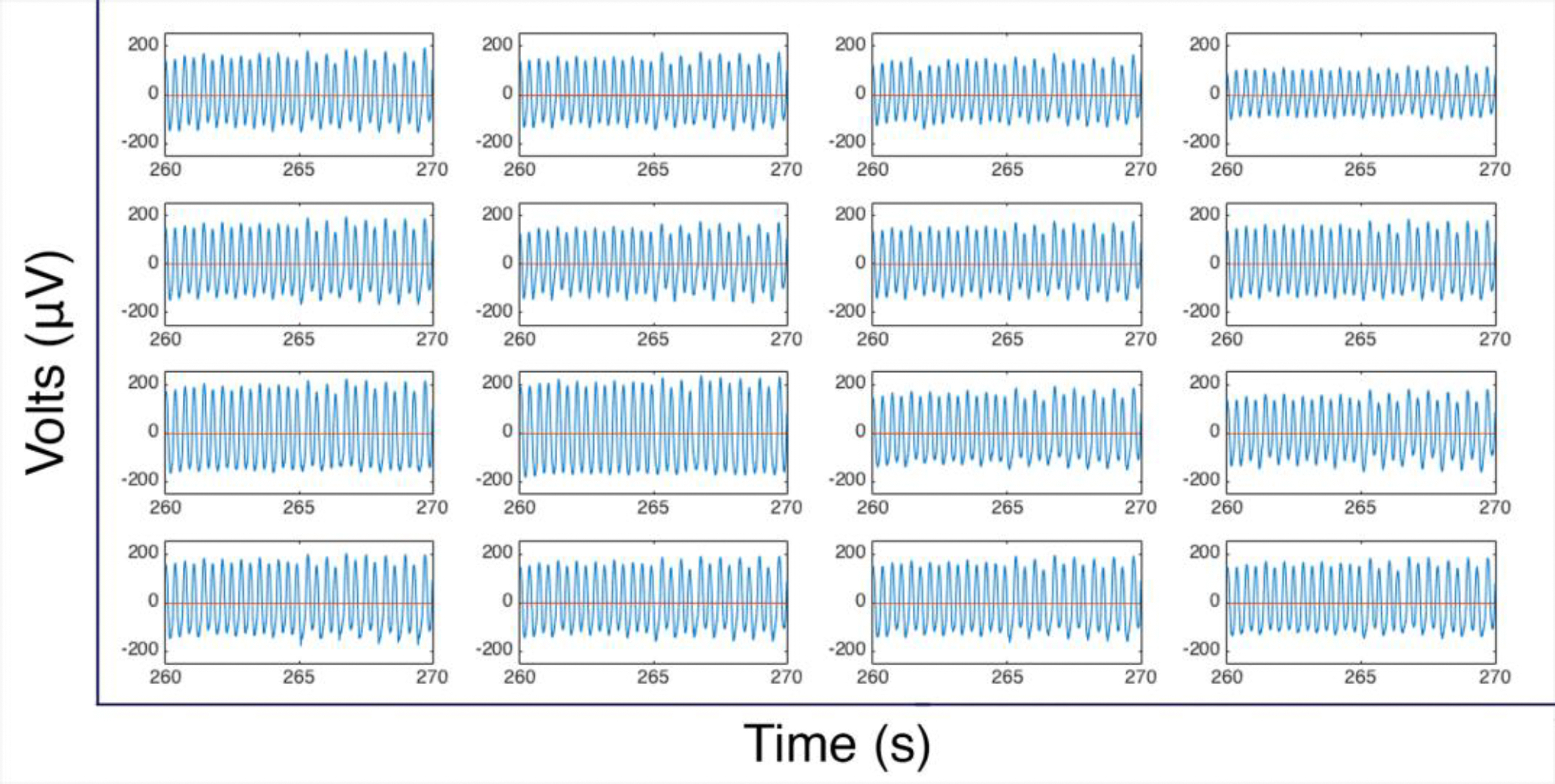
Electrical potentials acquired with EKG measuring system. Raw data acquired at all 16 channels laid out in the same configuration as is seen in the electrode board used in this study.

To aid in assessing the EKG properties of our fabricated constructs, a Cole-Parmer Stable Dry Temp Dry Block Heater (Vernon Hills, IL) was set to 37°C to maintain the tissues during the testing period (Figure 2). The tissue construct was removed from the culture plate and placed on top of the electrode board with the top surface facing down for maximum cell to electrode contact (Figure 2). The electrical potential of each tissue construct was measured for up to 30 minutes, and the data was stored in 1 minute RHD file recordings (Figure 3). We tested 3 tissue constructs using this approach and present representative tracings in the results section.

### 2.8. Data Analysis

Once the data collection process had concluded the raw data was run through an open-source m-file provided by Intan Technologies to import the data into MATLAB (MathWorks, Natick, MA) for analysis. Only files that displayed consistent readings as well as minimal artifacts from acoustic or electronic sources were chosen as representative of the desired data set. The chosen files were then run through a custom m-file to retrieve the cross-correlation values between the signals of each channel and the chosen reference channels. This information along with the sampling rate was used to construct time delay tables, which were used to generate the electrical impulse propagations maps. The electrical maps are made by assigning a color range to the values of the time delay tables, making it possible to visually express the impulse propagation from the reference channel to the 15 other channels with respect to time. The electrical potential of each data set was also evaluated using a second custom m-file, which allowed us to obtain the average wave amplitude, time to peak, and relaxation time (Figure 3).

## 3. Results

**Figure 4.**
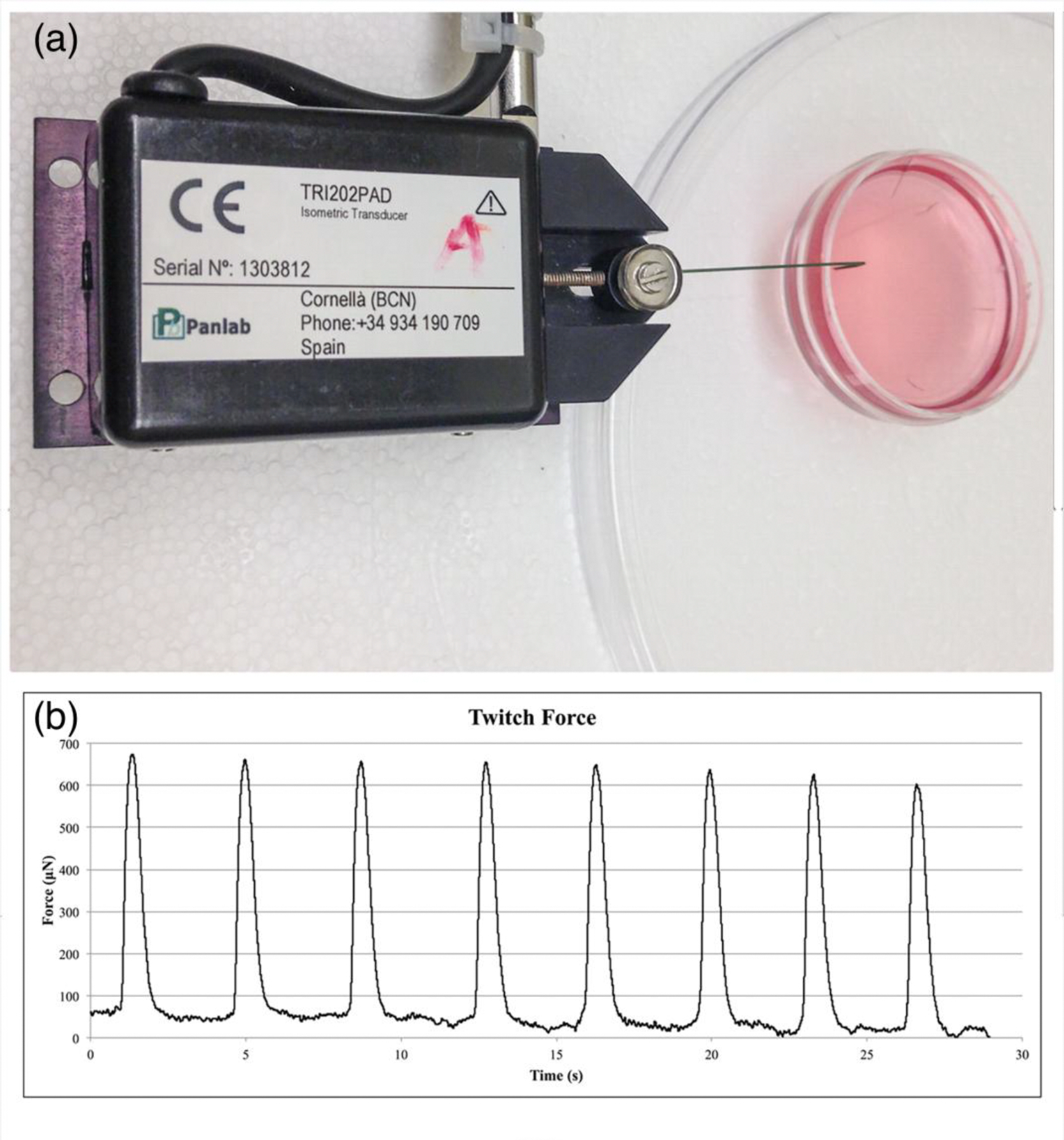
Contractile properties assessment. **(a)** Image showing the setup used to acquire the contractile properties of the fabricated tissues **(b)** Contractile properties of the cultured tissue with a plating density of 4 million cells per construct.

**Figure 5.**
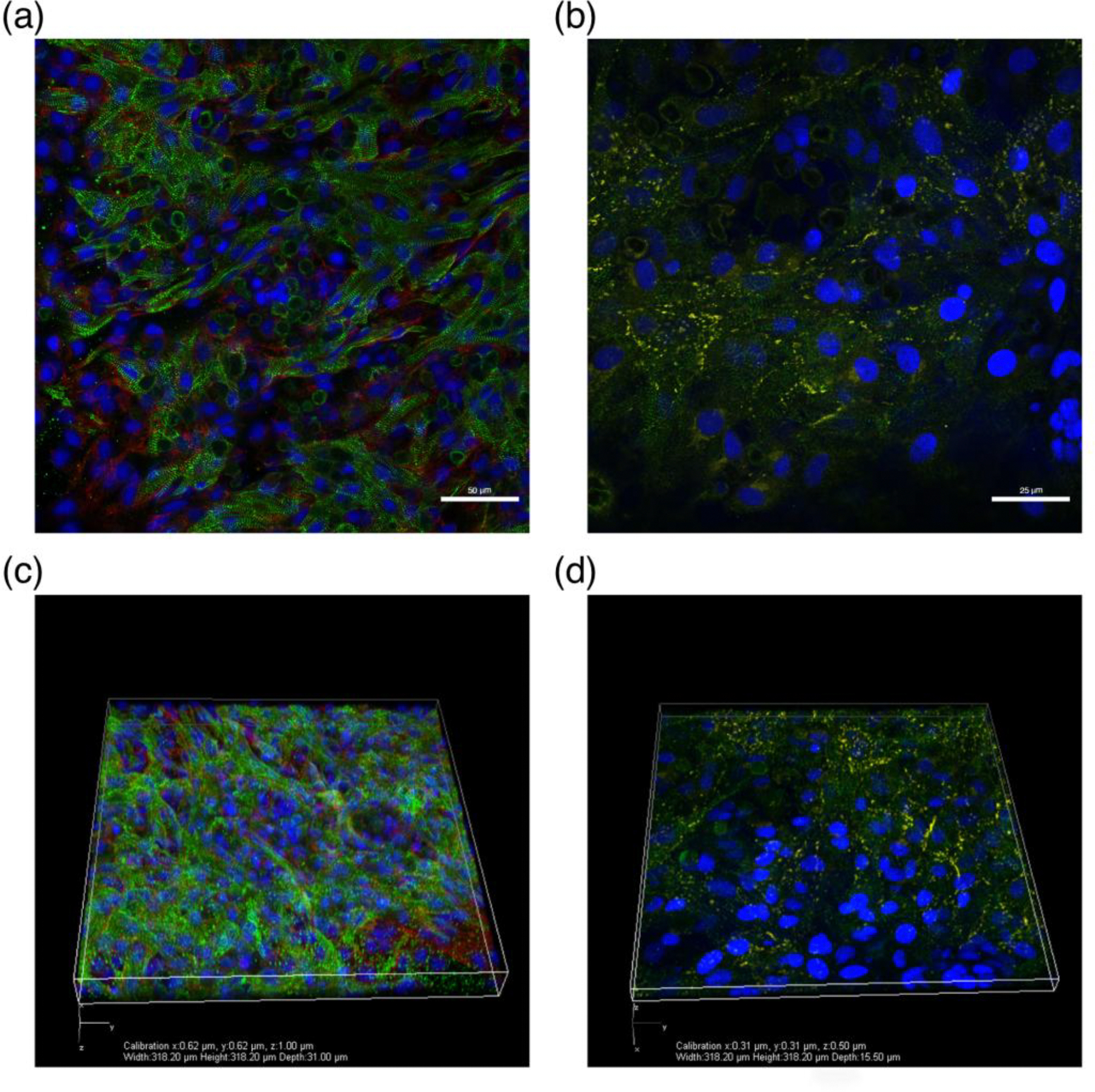
Immunohistological assessment of 3D-AHM. **(a)** Immunofluorescent image showing positive staining for α-actinin (green) and collagen type I (red); **(b)** Immunofluorescent image showing positive staining for α-actinin (green) and connexin 43 (yellow); **(c)** Immunofluorescent z-stack image showing positive staining for α-actinin (green) and collagen type I (red); **(d)** Immunofluorescent z-stack image showing positive staining for α-actinin (green) and connexin 43 (yellow).

During culture of 3D-AHMs, we observed that the delamination began approximately after 3 days, which led to the formation of the desired shape with the minutien pins as anchor points. The delamination process is directed by the contractions in the cell layer that initially forms on the surface of the fibrin gel. Within 4 to 5 days, the tissues are formed, contracting more synchronously, and ready for testing. Contractile force was measured and found to be approximately 600 μN for tissues that have been in culture from 6 to 7 days after initial cell plating (Figure4). Furthermore, immunostaining showed the presence of many cardiac markers (Figure 5).

Once the tissues had formed (4 to 5 days in culture), our novel system was used to evaluate the electrophysiological properties of the 3D-AHM. With reference channels at each corner of the electrode board, the chosen representative files for the acquired data were used to retrieve the cross-correlation values between the signals of each channel and the chosen reference channels. Cross-correlation yields a peak where the signals overlap, which corresponds to the number of samples the signals are lagging by with respect to the reference. This information along with the sampling rate was used to determine the time delays between the signals, which we found to be in the range of 0–38 ms.

**Figure 6.**
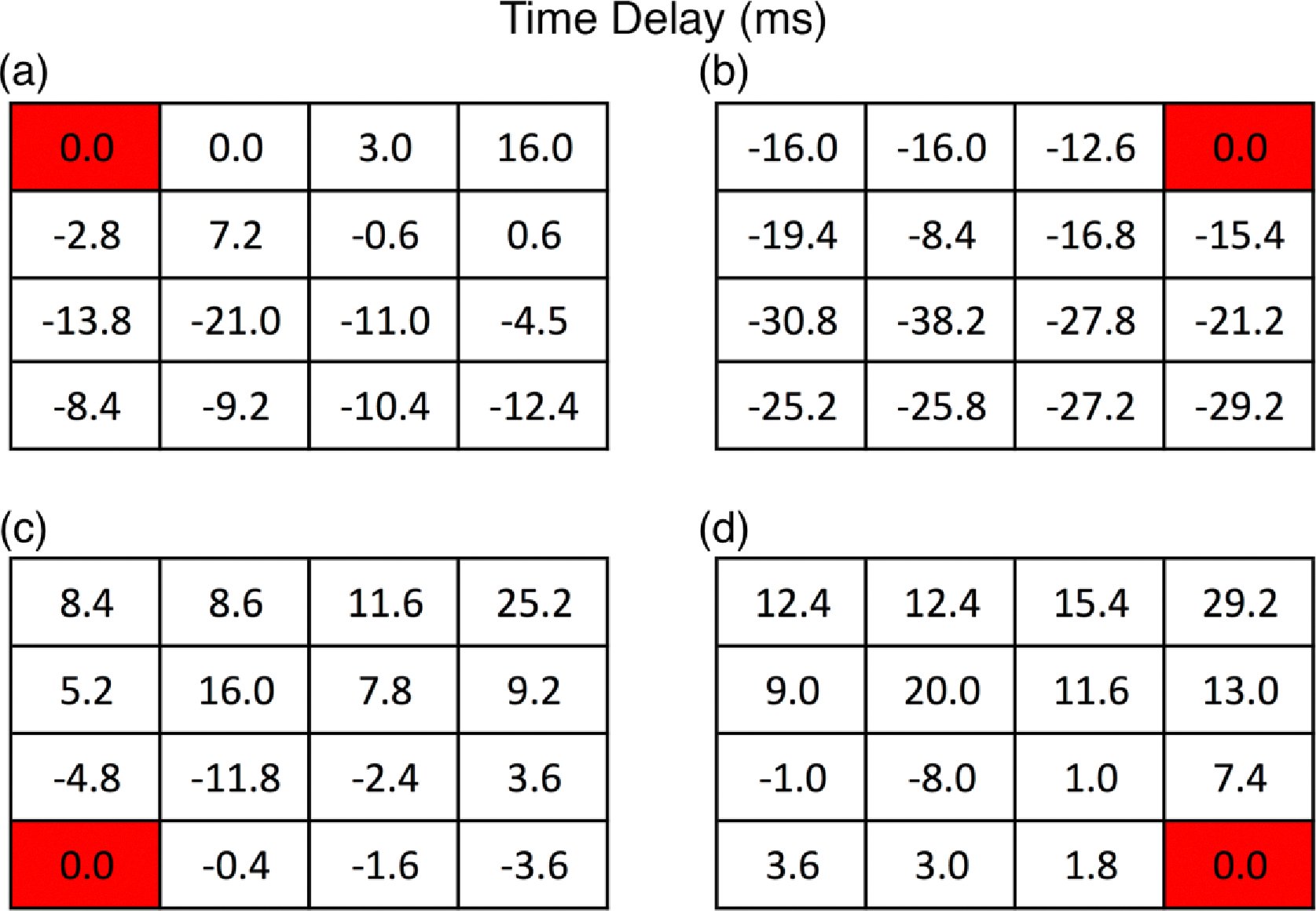
Time delay tables of 3D-AHM impulse propagation. **(a)** Time delay table constructed using channel 1 as the reference; **(b)** Time delay table constructed using channel 4 as the reference; **(c)** Time delay table constructed using channel 13 as the reference; **(d)** Time delay table constructed using channel 16 as the reference.

**Figure 7.**
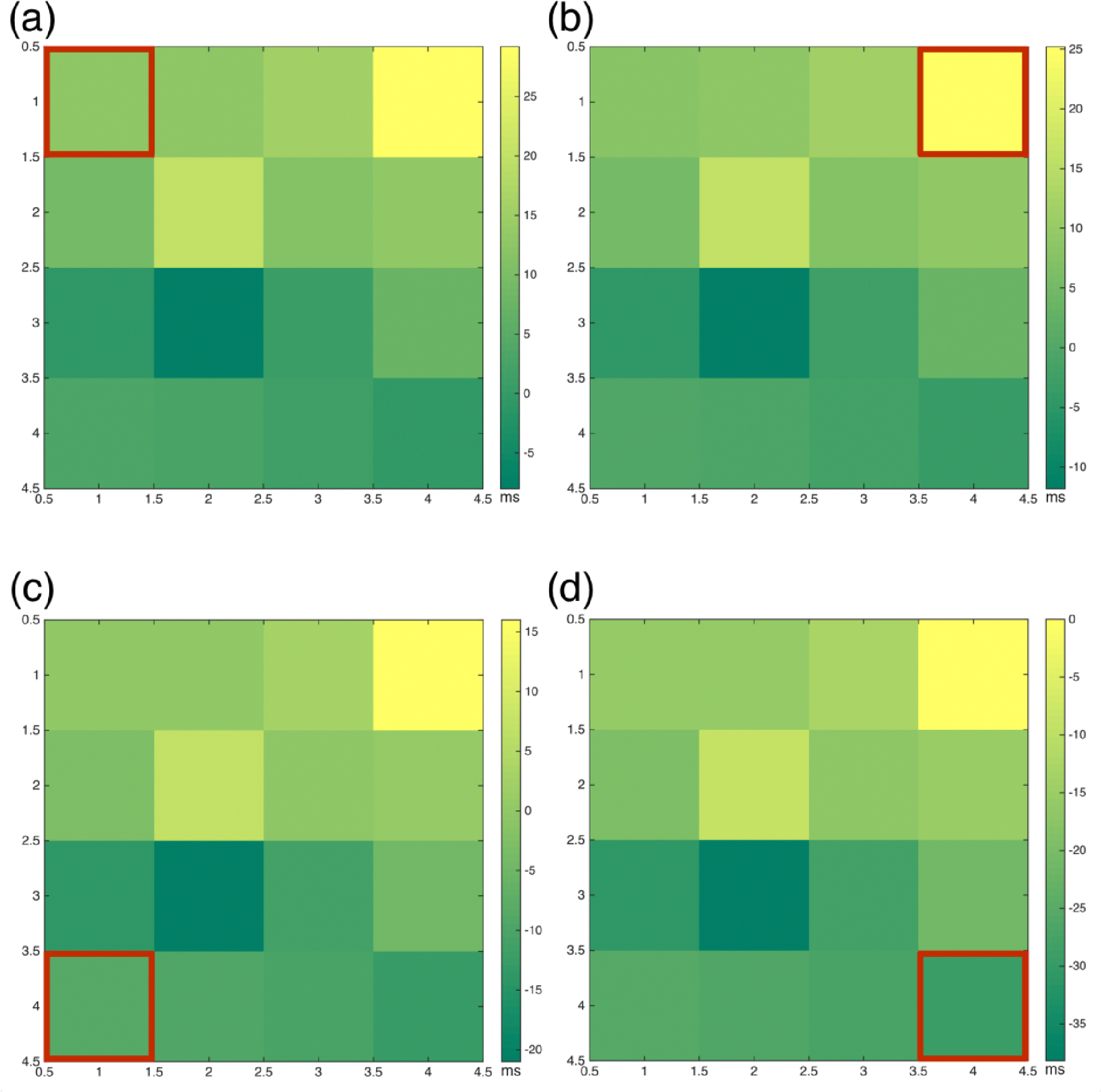
Impulse propagation electrical maps. **(a)** Electrical impulse map constructed using channel 1 as the reference; **(b)** Electrical map of the time delay between the different channels when electrode 4 was picked as the reference; **(c)** Electrical map of the time delay between the different channels when electrode 13 was picked as the reference; **(d)** Electrical map of the time delay between the different channels when electrode 16 was picked as the reference.

**Figure 8.**
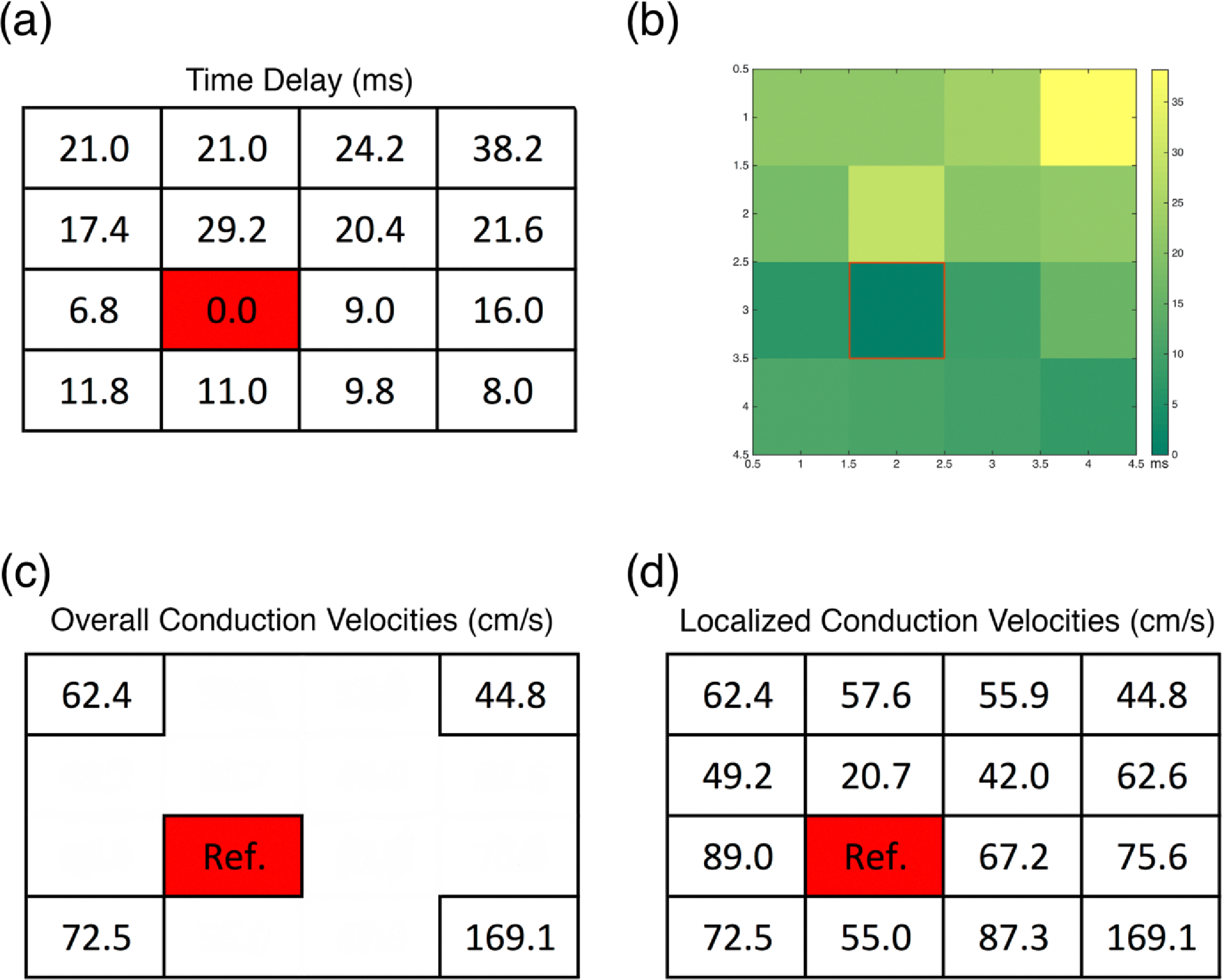
Time delay, electrical maps, and CVs with respect to channel 10. **(a)** Time delay between the different channels when electrode 10 was picked as the reference; **(b)** Electrical map of the time delay between the different channels when electrode 10 was picked as the reference; **(c)** Overall CVs calculated with respect to channel 10 **(d)** Localized CVs calculated with respect to channel 10.

After time delays were determined, we constructed tables of the values respective to the location of the electrodes on the sampling board (Figure 6). Time delay tables were then used to illustrate the propagation of the electrical potential within the tissues by using electrical maps which also yielded information regarding signal origin within the tissue; channel 10 (Figure 7). With the data obtained from the time delays (Figure 8a), we were able to construct the impulse propagation map shown in Figure 8b.

Once the origin of contraction was determined the localized CVs corresponding to the 3D-AHM with respect to that channel were calculated. The calculations were based on the values acquired for the impulse propagation and the known distances traveled between the 16 channels. With this reference channel we were able to calculate overall CVs radiating from channel 10. We found the CV from the reference to the upper left corner of the board (channel 1) to be 62.4 cm/s, to the upper right corner (channel 4) 44.8 cm/s, to the lower left corner (channel 13) 72.5 cm/s, and to the lower right corner (channel 16) 169.1 cm/s (Figure 8c). We were also able to compute localized CVs in the range of 20–170 cm/s as shown in Figure 8d.

**Figure 9.**
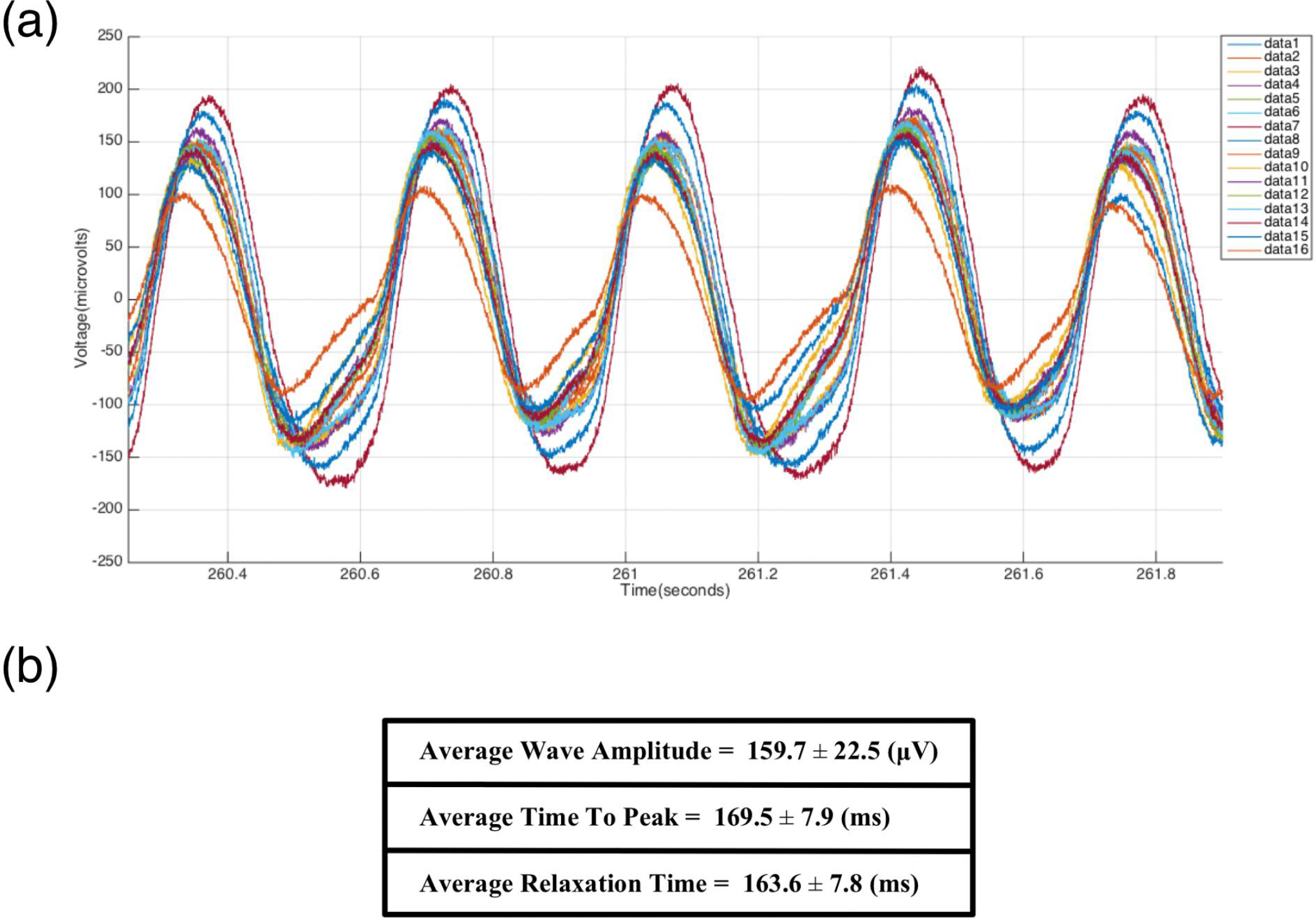
Peak analysis. **(a)** 10 second segment showing the waveforms for all 16 channels; **(b)** Average values calculated from the evaluation of the electrical potential signals of fabricated heart muscle.

The raw data was processed through a second custom m-file to perform peak analysis of the acquired data. We extracted 10s segments of raw data from all 16 channels (Figure 9a), and we were able to determine the average wave amplitude, time to peak, and relaxation time (Figure 9b). The average wave amplitude was found to be 159.7 ± 22 μV. The average time to peak was found to be 169.5 ± 7.9 ms. Finally, the average relaxation time was 163.6 ± 7.8 ms. Overall duration of the electrical potential cycle was calculated to be 360 ± 0.4 ms.

## 4. Discussion

The mammalian heart lacks the innate ability to self-repair damaged sections after acute myocardial infarction. The development of tissue equivalents that can repair or replace damaged sections of the heart has become an alternative paradigm where other treatments may fail. Cardiac tissue engineering has progressed to a degree where many different strategies may be employed to develop tissue constructs. However, current techniques concomitant with an appreciable deficit of adequate instrumentation have not yielded a facultative match to native heart muscle [3].

Our custom fabricated 16-electrode board, compatible with the RHD2000 Evaluation System from Intan Technologies, is capable of addressing concerns associated with previously devised methods. One such issue is fractionation of the waveforms and low amplitudes recorded by one research group [4]. This problem is avoided in our system due to a higher resolution during data acquisition. Another group using a separate commercially available system was able to obtain a narrow range of data compared to the data acquired by our system [15]. Lastly, a 32 channel direct contact system developed within our laboratory proved efficient in gathering all desired metrics [16], however, the pins in contact with the fibrinogen-cardiomyocyte construct caused some tearing at the tissue interface level. All propagation maps acquired during this study showed a trend of time increasing as distance from the reference increases. However, at specific channels the trend is not seen, leading to the conclusion that the tissue constructs have asynchronous impulse propagation at some points, which may be a result of cell agglomeration during the fabrication process.

Immunohistological assessment of our tissues was also performed to validate the development of cardiac muscle by observing markers commonly seen in 3D-AHM. Positive staining for α-actinin (green) reveals z-lines of cardiac myofibrils. Positive staining for collagen type I (red) indirectly shows the presence cardiac fibroblasts within the 3D-AHM. As well, positive staining for connexin 43 (yellow) illustrates evidence of electromechanical coupling within the tissue. Staining for, and demonstrating the presence of these markers verifies the development of heart muscle (Figure 5a–5b). Additionally, acquired z-stack images of the tissues with these specific markers show the three dimensionality of the constructs and some degree of tissue level organization (Figure 5c–5d). Moreover, a study, previously published by our lab on the optimization of our tissues, shows the presence of similar markers analyzed during this study as well as other cardiac markers. This process not only provides further insight into the characterization of our tissues, but also proves the presence of other types of cells that are needed to support the development of 3D-AHM [19].

Understanding the electrical impulse propagation within the myocardium is crucial for proper tissue development. It is these impulses that regulate the cardiac cycle and the synchronized contraction of the heart. The software interface provided by Intan Technologies allowed us to modify the bandwidth and amplifier-sampling rate to obtain the best signal possible. Our system recorded the electrical potentials of the constructs for up to 30 minutes, which were automatically broken up into separate 1-minute RHD files. These files were run through an open source MATLAB file to extract the data and perform any necessary analysis. This allowed us to look at each individual channel to obtain time delays, impulse propagation, CVs, and perform peak analysis.

Computation of the time delay was accomplished by obtaining the maximum value of the cross correlation function, which points to the instant where the signals are best aligned yielding the number of samples the signal is lagging by with respect to the chosen reference. The properties of cross correlation minimize errors in the calculation process, despite the presence of noise. The obtained sample lag from each channel multiplied by the sampling rate used (5.00 kS/s) allowed us to obtain the time delay between each signal and the reference. The acquired time delays were used to construct tables that displayed the values with respect to each reference as can be seen in Figure 6. Next, we constructed electrical maps to illustrate impulse propagation across 3D-AHM with respect to the chosen reference (Figure 7). We discovered that the tissues exhibited a trend in impulse propagation, however asynchronous characteristics were present at certain regions of the constructs, leading to an overall non-uniform electrical dispersion. The incongruity can be explained by the presence of cardiac pacemaker cells, which can cause contraction to start spontaneously at different regions of the 3D-AHM. Additionally, uniform contraction depends on the proportion of fibroblasts, smooth muscle cells and endothelial cells [19]. Seeing that these supporting cells have a faster proliferation rate than cardiomyocytes, this may also justify the discrepant propagation of the electrical impulse. Additionally, the absence of uniformity may be explained by the lack of electrical or cyclical mechanical stimulation, which has previously demonstrated a penchant for increasing membrane polarization of both N-cadherin and connexin 43 junctions leading to a more highly coordinated contraction [14]. Both stimuli paradigms will be implemented in subsequent studies to better condition 3D-AHM during the culturing process. In the next step of the process, we evaluated both the electrical maps constructed and the time delays calculated to determine the approximate origination location of the signal for the construct, which pointed to channel 10 as that origin. A time delay table and electrical map were constructed using this channel as a reference (Figure 8a–8b). From the time delay tables we were also able to obtain the CVs of the 3D-AHM (Figure 8c). The previous analysis of the electrical potential signals for each channel along with known distances between channels were used to compute the overall and localized CVs over the total area of the construct, which could not be achieved by systems used by other research groups. In previous studies, a research group found the neonatal ventricular electrical signal propagation to be 21.84 ± 1.48 and that of adult ventricles to be 31.69 ± 4.44 cm/s [4]. Other researchers reported that their bioengineered cardiac constructs exhibited CVs of 9.35 ± 0.27 cm/s and 11.89 ± 0.46 cm/s when enriched. Another group showed an average impulse propagation of 8.6 ± 2.3 cm/s in their cardiac constructs [5]. We observed overall and localized CVs to be in the range of 20–170 cm/s for 3D-AHM. Our calculated values differ from those found in literature, which may be explained by the usage of different methodology during the fabrication process. Additionally, the majority of studies performed in the past make use of ventricular cells, while we use cells found in both the atria and the ventricles. The contraction rate of atrial cells has been found to be 13–100 bpm and that of ventricular cells to be 14–42 bpm [20]. The wider range seen with atrial cells can be attributed to the presence of cardiac pacemaker cells, which have a contraction rate of 80–100 bpm. It is the presence of these faster beating cells in our tissues that can lead to higher CVs than those found elsewhere. As shown in a previously developed study, cells that aggregate during culture and that beat faster than other cells become the pacemakers and set the rhythm for contraction [21]. Moreover, our previous study [16], determined that mechanical movement of the construct during contraction did not account for the high CVs supporting our belief that distribution of different cell types across our tissues has the greatest influence over the CVs. In order to determine the validity of our hypothesis, studies of the electrophysiological properties of 3D-AHM fabricated with only ventricular cells need to be performed in the future.

Once all possible metrics regarding electrical impulse of the constructs were acquired, the files were processed using a secondary custom MATLAB program to perform peak analysis of the acquired raw data. From our tests we calculated the average wave amplitude to be 159.7 ± 22 μV. Bursac et al. acquired average amplitudes of 260 ± 90 μV for their regular constructs and 430 ± 140 μV for enriched constructs [4]. Our values are lower most likely from inverting the constructs onto the surface of the electrode board, possibly leading to some damage and/or loss of function. We also found the average time to peak (169.5 ± 7.9 ms), average relaxation time (163.6 ± 7.8 ms), and overall duration of the electrical potential (360 ± 0.4 ms). This overall interval duration is longer than the duration of the QRS complex seen in normal rats (23 ± 5 ms) [22]. This discrepancy can be explained by the difference in contractile function between 3D-AHM and native rat heart, which we hope to close over time. Although, 3D-AHM shows the formation of three-dimensional cardiac tissues, further improvements are necessary to better resemble the functional properties of the native heart muscle.
